# Supplementary material for: Patterns of Intron Gain and Loss in Fungi
Source: PLoS Biol. 2004 Nov 30;2(12):e422. doi: 10.1371/journal.pbio.0020422 (PMC532390; doi:10.1371/journal.pbio.0020422)
Supplement: Table S1 — Also available at http://genes.mit.edu/NielsenEtAl/. (4.3 MB ZIP). [file pbio.0020422.st001.zip › NielsenEtAl/html/288.html]

AN1460.1.NCU03369.1.MG02400.1.FG05168.1


```
 CLUSTAL W (1.82) Multiple Sequence Alignments - Introns Inserted


Sequence 1: NCU03369.1	304 aa
Sequence 2: MG02400.1	380 aa
Sequence 3: AN1460.1	310 aa
Sequence 4: FG05168.1	356 aa
Alignment Length: 388 aa
Number Identitical Residues: 182 aa
Alignment Score (without introns) 8741


MG02400.1 	MKSISTILIPIIVGLCSVFLFRDRIDDLTATLK~KYYNDNYISTTATHEPVNALPRTTTA
NCU03369.1	---------------------------------~--------------------------
FG05168.1 	---------------------MEYDFERLAARN0TSFKDTTTSTFEVLKTLTPLSYIYSD
AN1460.1  	---------------------------------~--------------------------
          	                                                            

MG02400.1 	TDQQPEEQEPHLYATEMSVPRTIRKVFEAIEQAEGAGARVRRSIGTPALRHFSPFLMLDH
NCU03369.1	----------------MSVPRLIRKIFLATEQAEGAGARVRRSIGTPQLRNFSPFLMLDH
FG05168.1 	KITDQQKDNHHLYATEMSVHRAIRKVFLAVEQSEGAGARVRRSIGTPQLRNFSPFLMLDH
AN1460.1  	----------------MSVPRAIRTVFLAIEQAEGAGARVRRSIGTPKLRNFSPFLMLDH
          	                *** * **.:* * **:************** **:*********

MG02400.1 	FSIKPGAGFPDH2PHRGQETITYLLTGAVDHEDFAGNKGTIEAGDLQFMTAGRG~IMHAE
NCU03369.1	FTIRPGSGFPDH~PHRGQETITYLLSGGVDHEDFAGNKGTIEAGDLQFMTAGRG~IMHAE
FG05168.1 	FSVKPGAGFPDH~PHRGQETITYLLEGGMDHEDFAGNRGTLSAGDLQFMTAGKG1IVHAE
AN1460.1  	FTIGKGAGFPDH~PHRGQETITYLLSGGVDHEDFAGNKGTIGPGDLQFMTAGKG~IMHAE
          	*::  *:***** ************ *.:********:**: .*********:* *:***

MG02400.1 	MPRQNPDGSANVGLQLWVDLPTHLKSCEPRYRDLRASEIPRADVDEGKVSVKIISGQSHG
NCU03369.1	MPRQNPDGSANVGLQLWVDLPKELKSCEPRYRDLKAQEIPTVETDEGRVKIKVISGKSHG
FG05168.1 	MPQQNADGSANIGLQLWVDLPKELKACEPRYRDLKAPEIPIAKIDDDKVTVKVISGQSHG
AN1460.1  	MPHENEDGSPNVGMQLWVDLPKKLKMCEPRYRDLRASEIPVATVDQGRVTVKVISGQSHG
          	**::* ***.*:*:*******..** ********:* *** .  *:.:*.:*:***:***

MG02400.1 	VDSVKDLAYTPVWILDVTIQPGGKISQPLPEGWNAFAYTLDGGDVILRDGAGTGARTVPQ
NCU03369.1	VDSVKDLAYTPVWILDVEVKPGGKVVQEVPEGWNAFAYTLEG-TAIFSSGKDGEKRTVEQ
FG05168.1 	IDSVKDLAYTPVWFLDIEIQPGGKITQPLPPNWNAFAYTLEGQVIV---GNDDQKRVVEQ
AN1460.1  	VDSVRDLAYTPVWLLDVAIKPGGRITQILPVGWNAFAYTLAGTTVF---GSNNSTQLVKQ
          	:***:********:**: ::***:: * :* .******** *   .   * .   : * *

MG02400.1 	YHMVVFEPKGEIVVAEVDAGADQAARFVLVAGTPLDQPVVQHGPFVVNSKEEIYKAIFDF
NCU03369.1	FHNVVFEPQGEAVYAEVDAGAEKPARFVLVAGTPLDQKVVQYGPFVLNSQEEVYQAFVDY
FG05168.1 	YHNIVFEPEGDVVHFEVDAGATKSARVAIIAGTPLDQPVIQYGPFVLTSKEDVAKALFDY
AN1460.1  	FHNVVFEQSGDHVEASVPDNAEEDSRFILVAGQPLDQKVVQYGPFVLTSQEEVYQAMLDY
          	:* :*** .*: *  .*  .* : :*. ::** **** *:*:****:.*:*:: :*:.*:

MG02400.1 	QTHSNGFERAKGWES~EIGKSMRD--------
NCU03369.1	QTHSNGFERAQGWRS~EIGKSMRH--------
FG05168.1 	QTHSNGFERAEDWQS~EIGKSMVG--------
AN1460.1  	QTASNGFERVRGWEK1YVWIIMMTTSNTSPHS
          	** ******...*..  :   *  ::.::. :
```
